# Accuracy of obesity indices alone or in combination for prediction of diabetes: A novel risk score by linear combination of general and abdominal measures of obesity

**DOI:** 10.22088/cjim.13.2.2

**Published:** 2022

**Authors:** Karimollah Hajian-Tilaki, Behzad Heidari

**Affiliations:** 1 Department of Biostatistics and Epidemiology, School of Public Health, Babol University of Medical Sciences, Babol, Iran; 2 Social Determinants of Health Research Center, Health Research Institute, Babol University of Medical Sciences, Babol, Iran; 3 Department of Internal Medicine, Ayatollah Rouhani Hospital, Babol University of Medical Sciences, Babol, Iran

**Keywords:** Body mass index, Waist circumference, Waist-to-height ratio, Waist-to-hip ratio, Diabetes mellitus, optimal combination

## Abstract

**Background:**

The predictive power of obesity measures varies according to the presence of coexistent measures. The present study aimed to determine the predictive power of combinations of obesity measures for diabetes by calculation of a linear risk score.

**Methods:**

Data from a population-based cross-sectional study of 994 representative samples of Iranian adults in Babol, Iran were analyzed. Measures of obesity including waist circumference (WC), body mass index (BMI), waist–to–height ratio (WHtR), and waist to hip ratio (WHR) were calculated, and diabetes was diagnosed by fasting blood sugar >126 mg/dl or taking antidiabetic medication. Multiple logistic regression model was used to develop a logit risk score based on BMI, WC, WHtR, and WHR. The ROC analysis was applied to determine the priority of every single index and combined logit score for the prediction of diabetes.

**Results:**

All four measures of general and abdominal obesity were predictors of diabetes individually in both sexes (P=0.0001). Calculation of risk score for a combination of all measures use full model improved predictive power. Adjustment for age resulted in further improvement in diagnostic power and combined novel risk score differentiated individuals with and without diabetes with an accuracy of 0.747 (95%CI: 0.690-0.808) in men and 0.789 (95%CI: 0.740, 0.837) in women.

**Conclusion:**

These findings indicate that the simultaneous calculation of age-adjusted risk score for all measures provides stronger diagnostic accuracy in both sexes. This issue suggests the calculation of combined risk scores for all obesity indices especially in a population at borderline risk.

General and abdominal obesity are major risk factors for the future development of hypertension, diabetes mellitus (DM), metabolic syndrome (MetS) and cardiovascular disease (1-2). These conditions are potentially linked with abnormalities in lipid profiles as well (3). The worldwide epidemic and global burden, economic costs, disability and the loss of lives because of obesity will remain high both in the developing and industrial countries (4-8). Obesity continues to have an increasing impact on the global health burden not only among adults but also in children and adolescents (9-11). It has been established that obesity, in particular, abdominal obesity is the main component of MetS (12-14). However, several other measures have been also recommended for evaluation of obesity and central obesity, such as body mass index (BMI), waist circumference (WC), waist to height ratio (WHtR) and waist to hip ratio (WHR) which are most often used in clinical practices.

Among these measures, the WC as a simple unidimensional measure has been further considered in the definition of MetS by International Diabetes Federation (IDF) (12), National Cholesterol Education Program (NCEP) Adult Treatment Panel III (ATP III) (13) and American Heart Association (AHA)/National Heart, Lung, and Blood Institute (14) as well as in Iranian National Committee of Obesity (INCO) but with different cutoff values (15). It has been acknowledged that all of the above anthropometric measures are also linked with insulin resistance and type 2 diabetes (16). Nonetheless, the advantage superiority and the optimal cutoff point values of each of these parameters over each other for the prediction of diabetes vary according to sex and ethnicities and so remain to be determined (17-18). 

Hence, a single measure may have insufficient potential to predict diabetes in subjects with or without general or abdominal obesity (19). For these reasons, a few studies have investigated the potential of combinations of these measures in the assessment of mortalities in patients with coronary artery disease. In a longitudinal study of Chinese adults, two indices of BMI and WC have provided a higher predictive ability for cardiovascular risk mortality as compared to each of them alone, particularly in women (20). In contrast, collaborative analysis of 58 prospective studies in developed countries, the separate or combined association of BMI, WC, and WHR did not importantly improve cardiovascular disease risk prediction in people of developed countries when additional information is available for systolic blood pressure, history of diabetes, and lipids after adjustment for age, sex, and smoking status, baseline blood pressure, history of diabetes and lipids (21). 

However, in the Asian population, in particular Iranian adult people, the predictive power of cardiovascular risk and diabetes for these measures alone or in combination has not been established yet. Thus, this study was aimed to develop a risk score of optimal combinations of obesity and central obesity measures in predicting DM.

## Methods

Study Subjects and Data collection: The data of this study were extracted from a population-based cross-sectional study of cardiovascular risk factors and MetS in Babol, a geographic region located in the North of Iran and the South of the Caspian Sea. A representative sample of 1000 adult population aged 20-70 years was recruited using a two-stage cluster sampling technique. The allocated sample size is enable to detect a standardized difference of 0.12 in calculated risk score between individuals with and without diabetes with 95% confidence level and 80% power. The six participants were excluded in the analysis because of the presence of missing data and thus the analysis was carried out with 994 individuals. 

The details of the sampling procedure and inclusion criteria have been explained elsewhere (22). In brief, the individuals with recent severe cardiovascular arteritis (CVA), major physical abnormality, pregnant women at second and third trimesters and those with less than 10 h fasting were excluded from the study. All demographic data such as age, gender, education, and also anthropometric measures of weight, height, WC, waist and hip circumferences were measured by trained nurses at home visit with standard methods. The weight was measured using a digital scale nearest to 0.1 kg with light clothes. The height was determined nearest to 0.1 cm using a stadiometer without shoes while the subjects stand near the wall. The WC was measured by a tape measure at the level of the midpoint between the iliac crest and the lower border of the tenth rib. Hip circumference is a measurement of the hip, using a tape measure, to assess the spatial distance between each corresponding hip bone in proportion to the buttocks. 

All subjects were invited to the central lab of Ayatollah Rouhani Hospital in taking blood samples during morning for10-12 hours overnight fasting. Fasting blood sugar (FBS) was measured by the standard method. Diabetes mellitus (DM) was diagnosed by FBS ≥126 mg/dl or self-reported data or taking antidiabetic medication. The BMI was calculated by weight in kg divided to height in m2 (kg/m2). The WHtR was computed as the ratio of WC to the height and the WHR was calculated as WC divided by HC in all participants. The calculated BMI was categorized as underweight (BMI<18.5), normal weight (BMI: 18.5-24.99), overweight (BMI:25-29.99) and obese (BMI>=30). For WC the cutoff point of 88 cm for women and 102 cm for men were used. All individuals had given written consent before participating in the study and the study protocol was approved by the Institutional Ethical Board of Babol University of Medical Sciences (Ethical code: IR.MUBABOL.Rec.1400.0660).

Statistical Analysis: The data were analyzed using the SPSS software Version 18. In the univariate analysis, the descriptive statistics for categorical variables were calculated as frequency and percentage and the quantitative variables as mean±SD. The binary outcome variable was FBS ≥126 mg/dl or taking antidiabetic agents defined as diabetes mellitus and all the anthropometric measures of obesity and abdominal obesity were independent variables. The age was considered as potential confounder and gender as modifier. In bivariate analysis, the means of anthropometric measures were compared between the two groups of participants with and without DM according to gender. 

The independent sample t-test was used for comparison between the two groups. In multivariate analysis, we developed a combination of risk scores based on BMI, WC, WHtR, and WHR for predicting DM using multiple logic regression models. Two different methods were used for the selection of variables. First, we used a forward stepwise method to select potential independent variables, and then we applied a full model consisted of all four obesity and abdominal obesity indexes according to sex. Based on logistic regression coefficients of full model, the risk score was calculated as logit p = b_0_ + b_1_BMI + b_2_ WC + b_3_WHtR + b_4_ WHR. Additionally, we adjusted the logit score in the full model by age according to the sexes. Finally, we applied receiver operator characteristic (ROC) analysis to calculate the diagnostic accuracy of the four anthropometric measures scores, alone or in a newly-developed combination of logit score for DM by estimating the area under the curve and its 95% confidence interval.

## Results

Characteristics of the study sample are presented in table 1 according to sex. The mean age (SD) in men and women were 43.6±14.3 and 41.9±12.6 years, respectively. The level of education in the majority (57.3%) of the participants was in high school or higher and it was significantly higher in men than women (65% vs 51.1%, P=0.001). Diabetes was found in 15.3% of men and 14.8% of women (P=0.65). While women had a significantly higher frequency of abnormal WC and BMI (P=0.001). 

**Figure 1:**
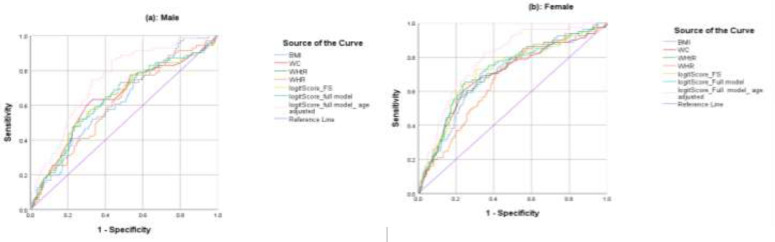
ROC curves of obesity and abdominal obesity measures and combined logit scores of different model selection (panels (a) for men and (b) for women)

Table 2 shows the results of regression coefficients of obesity indexes, SE, and Wald statistics with the stepwise forward method of variable selection in the logistic regression model. In men, only the WHtR remained at a significant level in age model and conditional on WHtR in the model, other indices remained non-significant. But for women, BMI and WHR remained significant using forward variable selection. Additionally, the coefficient logistic risk score of full model was also estimated for men and women (Men: logit P= - 5.264 + 0.011 BMI + 0.006 WC + 2.808 WHtR +1.221 WHR; Women: logit P = - 7.674 + 0.096 BMI - 0.026 WC + 2.881 WHtR + 4.424 WHR). The age adjusted logit risk score of full model was also calculated for both sexes (Men: logit P=-7.806+0.026BMI+0.085WC-11.393WHtR+0.526WHR+0.065 age; Women: logit P=-10.257+0.134BMI+0.008WC-6.199WHtR+4.808WHR=0.072 age). Table 3 indicates that all anthropometric measures and both the calculated logistic risk score are significantly higher in individuals with diabetes than in those without diabetes in both sexes (P= 0.001). Table 4 and figure 1 display the diagnostic accuracy of each anthropometric measure alone and the logistic risk score by three methods of variable selection. In forward selection, for men, the accuracy (AUC) of logistic risk score based on WHtR is identical to that of WHtR raw data alone. In contrast to women, the combined risk score based on the BMI and WHR produced higher accuracy than each marker alone. The additional gain on accuracies of the combined risk score of the full model was very little for both sexes. However, in the full model of all four anthropometric measures, when the logit score was adjusted by age, the diagnostic accuracy quietly improved compared with the unadjusted age in both sexes.

**Table 1:** The demographic and clinical characteristics of study samples according to sex

**Characteristics**	**All**	**Men**	**Women**	**P-value**
Age (Mean±SD, year)	42.7±13.4	43.6±14.3	41.9±12.6	0.06
Age group 20-39 y 40-59 >=60	428(43.1) 429 (43.2) 137 (13.8)	181 (40.4) 194 (43.3) 73 (16.3)	247 (45.2) 235 (43.0) 64 (11.7)	0.08
Educational level <High school High school or higher	424 (42.7) 570 (57.3)	157 (35.0) 291 (65.0)	267 (48.9) 279 (51.1)	0.001
DM Without DM With DM	841 (84.7) 152 (15.3)	376 (84.1) 71 (15.9)	465 (85.2) 81 (14.8)	0.65
WC (cm) Normal WC (Men<102, women<88) Abnormal WC (Men > 102, Women > 88)	565 (57.1) 426 (42.9)	342 (76.3) 106 (23.7)	226 (41.4) 320 (58.6)	0.001
BMI (kg/m^2^) <18.5 18.5-24.9 25-29.9 >=30	13 (1.3) 322 (32.4) 373 (37.5) 286 (28.8)	5 (1.1) 185 (41.3) 177 (39.5) 81 (18.1)	8 (1.5) 137 (25.1) 196 (35.9) 205 (37.5)	0.001

**Table 2:** The logistic regression risk score coefficients using stepwise forward variable selection using anthropometric indexes in predicting DM

**Variables**	**Coefficients (β)**	**SE(β )**	**Wald statistics**	**P-value**
**Men** Constant WHtR	-4.47 5.02	0.84 1.47	28.10 151.46	0.001 0.001
**Women** Constant BMI WHR	-7.66 0.09 3.81	1.34 0.02 1.51	32.67 17.84 6.36	0.001 0.001 0.012

Men: Logit p=-4.47+5.02 WHtR; Women: logit p=-7.66+0.09BMI+3.81WHR

**Table 3:** The mean SD of anthropometric measures and the computed risk scores in individuals with and without DM

**Anthropometric indexes**	**With DM** **Mean± SD**	**Without DM** **Mean± SD**	**P-value**
**Men** BMI (kg/m^2^) WC (cm) WHtR WHR Logistic risk score (Forward selection) Logistic risk score (Full model)˧ Logit risk score (Full model)˦	27.76 ±3.97 99.14 ±17.97 0.58± 0.12 0.94± 0.13 -1.554 ±0.589 -1.578±0.591 -1.578 0.591	26.19±4.97 92.48 ±13.69 0.54±0.08 0.91±0.08 -1.766±0.404 -1.798±0.406 -1.798 0.406	0.001 0.001 0.001 0.001 0.001 0.001 0.001
**Women** BMI (kg/m^2^) WC (cm) WHtR WHR Logit risk score (Forward selection) Logit risk score (Full model)˧ Logit risk score (Full model)˦	31.73± 5.73 98.62 ±14.73 0.63 ±0.09 0.87 ±0.09 -1.473± 0.676 -1.513±0.656 -1.222 0.883	28.13± 5.49 90.31±14.65 0.57± 0.09 0.83 ±0.09 -1.961±0.673 -2.008±0.676 -2.381 1.140	0.001 0.001 0.001 0.001 0.001 0.001 0.001

˧unadjusted by age

˦adjusted by age

**Table 4:** Accuracy of anthropometric measures alone and combined logit risk scores in predicting diabetes with respect to sex

**Anthropometric indexes**	**AUC (95%CI)**	**P-value**
**Men** BMI WC WHtR WHR Logistic risk score (Forward selection) Logistic risk score (Full model)˧ Logit risk score (Full model) ˦	0.621 (0.554, 0.688) 0.641 (0.556, 0.715) 0.636 (0.563, 0.710) 0.610 (0.540, 0.680) 0.636 (0.563, 0.710) 0.639 (0.567, 0.711) 0.747 (0.690, 0.808)	0.001 0.001 0.001 0.003 0.001 0.001 0.001
**Women** BMI WC WHtR WHR Logit risk score (Forward selection) Logit risk score (Full model)˧ logit risk score (Full mode)˦	0.692 (0.629, 0.755) 0.692 (0.626, 0.758) 0.703 (0.640, 0.766) 0.664 (0.603, 0.725) 0.715 (0.652, 0.778) 0.717 (0.652, 0.776) 0.789 (0.740, 0.837)	0.001 0.001 0.001 0.001 0.001 0.001 0.001

Full model logit risk score without age adjusted; ˦ Full model adjusted by age.

## Discussion

The results of this study indicate that all anthropometric measures of obesity such as BMI, WC, WHtR, and WHR individually can predict diabetes significantly in both men and women. However, performances of these indices vary according to sex. In men, despite a significant accuracy, predictive performances for all indices were low, whereas in women, all measures especially WHtR yielded acceptable predicted power individually with an accuracy of close to a good level. Furthermore, risk calculation for the combination of these indices together provided stronger predictive ability in distinguishing diabetic from nondiabetic individuals at a significant level in both sexes particularly in women. In this study, BMI, WC, WHtR and WHR simultaneously predicted diabetes type 2 and differentiated it from the non-diabetic population with accuracy 74.7% in men and 78.9% in women. In the female population of this study, the accuracy of BMI as an index of general obesity and WC as a measure of abdominal obesity was lower than WHtR which is considered as a measure of abdominal fat distribution, whereas in men accuracy of BMI and WHtR particularly WHR was lower than WC. Overall, these findings indicate that each obesity measure especially WHtR, in addition to individual predictive performance, can provide stronger predictive power in combination with other anthropometric measures. 

Anthropometric parameters that are easily measurable are frequently used for the identification of individuals who are at greater risk of cardiometabolic disease. In the present study, greater predictive power of WHtR is partly in agreement with the results of 3 studies from other Iranian populations in which WHtR yielded the highest predictive ability in both sexes, whereas, other measures exhibited equal ability in each sex (23, 24, 25. However, none of these studies calculated combined risk of anthropometric measures, only Zafari et al. (24) in their recent meta-analysis have found higher predictive value in both men and women when high values of BMI were combined with other anthropometric measures. Similarly, Lee et al. (26) found superiority of calculation of combined risk score for prediction diabetes.

Accordingly, the results of 4 previous meta-analyses, as well as a recent meta-analysis, has revealed stronger or equal accuracy of WHtR over WC and superiority of both WHtR and WC over BMI in predicting diabetes (27, 28, 29, 31). In one meta-analysis, WHtR was the best discriminator for hypertension, diabetes, and dyslipidemia in both sexes, whereas BMI had the poorest discriminative power for cardiovascular risk factors (28). Similarly, Ashwell et al. (30) found greater discriminative potential for WHtR and WC than BMI in predicting the adverse outcome by 4- 5% and 3% over BMI, respectively. Savva et al. (31) found a stronger association of WHtR than BMI with incident cardiovascular disease, cardiovascular disease mortality, and all-cause mortality in Asian than non-Asian. Also, in two cohort studies comprised of different ethnic populations (32, 33), the accuracy of WHtR and WC was higher in the Asian population. Nonetheless, Kidy et al. (34) in a bi-ethnic sample of South Asians and white Europeans found no superiority of any obesity measure to BMI across diverse ethnicities in the UK. Even, a recent longitudinal study of 9962 Chinese elderly people has shown stronger predicting power of BMI than WC and WHtR, both in men and women over a 4.6- year follow-up duration (35).

Contrary to our study, Ye et al. (36) in a recent longitudinal study found higher accuracy of BMI, WC, and WHR in men than in women. In this study baseline, BMI and WC as well, as the prevalence of overweight and obesity in men were significantly higher than in women. 

Conflicting results between different studies can be attributed to several factors including patient selection, study design, methods of data collection, characteristics of the study population such as age, sex, ethnicity, lifestyles. Furthermore, the prevalence of general and abdominal obesity, as well as diabetes, vary across diverse studies. Besides, the association between anthropometric measures and cardiometabolic risk factors can be confounded by the distribution of other associated factors of diabetes like family history, diet, level of physical activity and education (7-10). 

In the geographic region of the current study, diabetes and obesity are prevalent in the general population, particularly in women, even in adolescent and children (7-10), hence predictive power of anthropometric measure for diabetes is expected to be different from of other studies with different prevalence of diabetes and its risk factors. Thus, the combined score in our findings is useful for fast screening in clinical practices. With regard to the participants of the present study, the prevalence of diabetes was similar between men and women, but the prevalence of abdominal and general obesity in women was about two-fold greater than in men. As a result, the accuracy of anthropometric measures especially WHtR or WC in men was expected to be lower. However, the difference in accuracy decreased between women and men after the calculation of risk score for the combination of BMI, WC, WHtR, and WHR.

 The results of a cross-sectional study of obese men and women indicated that in women, all measures of body fat distribution except abdominal subcutaneous adipose tissue were associated with having ≥ 1 cardiometabolic risk factor, in which visceral adipose tissue was most strongly associated by OR = 5.77, whereas in obese men, the associations of body fat distribution and the presence of cardiometabolic risk factors were attenuated (37).These observations may indicate that factors other than obesity measure may also contribute to the development of diabetes in men, and thus may explain sex-difference accuracy in this study.

Nonetheless, the present study is cross-sectional and the association does not indicate causality. One major strength of this study is the simultaneous calculation of risk score for all anthropometric measures which in our knowledge has not been shown in any previous studies yet. This method provides greater power for prediction of outcome and thus may increase the predictive power of a variable that could not be yielded when used alone.

The findings of this study regarding risk calculation of anthropometric measure especially WHtR and WC are important clinically because risk score calculation provides a simple measure for recognizing individuals who are at higher risk of cardiovascular disease. A meta-analysis of 31studies found WHtR as a quantitative measure for cardiovascular risk regardless of the degree of general obesity, and a WHtR ≥ 0.5 was associated with 4.15 times risk of metabolic syndrome (38). Moreover, high WHtR is associated with an increased risk of hypertension, and also the best determinant of successful aging. In one study, WHtR was inversely associated with successful aging. In this study, every 0.1-unit increase in WHtR was associated with a 0.5 unit decrease in a successful aging index and the association was greater after surpassing age and sex (39).

This study might have some limitations. The cross-sectional nature of this study limits the interpretation of causality between obesity measures and diabetes and the reverse causality bias might be possible. In addition, the diagnosis of diabetes was based on FBS and/ or taking antidiabetic medication. The other criteria such as hemoglobin A1C might be useful for future study. Moreover, there are possibility of measurement errors in anthropometric measures. However, such measurement errors are non-differential with respect to diabetes status. Thus, it may cancel out and thus it cannot lead to distortion in estimating of risk score in comparison between two groups.

In conclusion, the findings of this study indicate that each anthropometric measure of obesity especially measures of abdominal obesity are predictors of diabetes individually. The particular calculation of simultaneous risk score for all anthropometric measures provides stronger accuracy for both sexes, especially in women. This issue suggests simultaneous calculation of risk scores for all obesity indices in subjects with anthropometric measures within borderline or even in ranges of upper normal limits.
